# Topics of Public Interest

**Published:** 1906-03

**Authors:** 


### TOPICS OF PUBLIC INTEREST.

### Army Medical Corps Examinations.

Preliminary examinations for appointment of Assistant Surgeons in the Army will be held on May 1, and July 31, 1906, at points to be hereafter designated.

Permission to appear for examination can be obtained upon application to the Surgeon General, U. S. Army, Washington, D. C., from whom full information concerning the examination can be procured. The essential requirements to securing an invitation are that the applicant shall be a citizen of the United States, shall be between twenty-two and thirty years of age, a graduate of a medical school legally authorised to confer the degree of doctor of medicine, shall be of good moral character and habits, and shall have had at least one year's hospital training or its equivalent in practice. The examinations will be held concurrently throughout the country at points where boards can be convened. Due consideration will be given to the localities from which applications are received, in order to lessen the traveling expenses of applicants as much as possible.

In order to perfect all necessary arrangements for the examinations of May first, applications must be complete and in possession of the Surgeon General on or before April first. Early attention is therefore enjoined upon all intended applicants.

There are at present twenty-five vacancies in the medical corps of the army.

### The Passing of a Surgeon.

### GEORGE RYERSON FOWLER.

### AN APPRECIATION.

By WALTER B. CHASE, M. D., Brooklyn.

THE untimely death of a great man startles the community. The passing away of Dr. George Ryerson Fowler brings to the medical profession and a large clientele a shock and a regret which is uppermost in the minds of a large number of people, and quickens their memory of those aspirations and qualities which made him great.

Dr. Fowler was preeminently the architect of his own fortune. The eminence he attained was neither accidental nor easy. Forced by circumstances to make his own way in the world, he struggled in the acquiring of his professional education against adverse circumstances, which would have disheartened most youths, the exercise of which schooled him in the development of those elments of character,—self-reliance, patience and persistence,—which showed so conspicuously in after life and fitted him for those arduous and exacting demands which raised him to so high a position in the world of science.

Not more was he noted in the general technic of surgical work than in the mastery of every detail in anatomy and pathology, those underlying attainments without which he never could have triumphed.

His grasp of surgical and pathological problems was only equaled by his tremendous power of concentration of thought and searching analysis of every problem which bore on the welfare of his patients. Those who knew him best were alone aware of his capacity for arduous labor. When others slept he was at work. His success was measured largely by his power of intense and prolonged application.

By these methods he not only became preeminently familiar with his professional work, but was proficient in modern languages, and familiar with the best literature of the times. For more than a quarter of a century he was an ardent admirer and follower of Lister's antiseptic and (later) aseptic surgery, and to faithfulness in which he carried out these details was one secret of his success. His ability as a surgeon was greatly enhanced by his thorough knowledge of the practise of general medicine years before he devoted himself to the exclusive practise of surgery. If many of the newly-fledged specialists of today had such an experience in the practise of general medicine, they would bring to their labors fitness which warrants better work than they are now prepared to do. One qualification which added to his name and fame was his marvelous power of rapid and accurate diagnosis. While others reached their conclusions by a series of exclusive reasoning, his quick grasp and intuition led him to immediate and correct deductions.

Another characteristic of the man as a surgeon was his marvelous resourcefulness. In those supreme moments of a surgeon's life—in the crisis of an operation on which hung the destiny of the patient—when everything depends on properly appreciating and promptly dealing with new or unexpected complications, he was at his best—cool and collected, he rose equal to the emergency.

If an ayalysis were to be attempted of his characteristics as an operator, the element of boldness would stand out prominent. Not the boldness of rashness, which might jeopardise the patient, but the boldness of conservatism, coupled with courage and rare judgment, which would do the right thing at the right time. His radicalism, as paradoxical as it may appear, was the conservative basis of his procedure.

As a contributor and author his writings were voluminous and commanded the attention of the profession at home and abroad. His growth as a surgeon was progressive and symmetrical. The demands for his services grew with every new attainment. His unostentatious confidence in himself inspired similar confidence in others, and so within and outside the medical profession his reputation grew and expanded.

This appreciation was recognised by his professional brethren. Called from one important hospital position to another, he was made examiner in surgery for the University of the State of New York, a position he held until the time of his death. The crowning effort of his eventful career consisted in an original and exhaustive work on surgery which is now in press, to be issued by the most progressive publisher of medical libraries in the world. His reputation as a surgeon and author was widespread. Not only was he known as authority on surgery in this metropolis, but throughout the United States, and in the best medical circles of foreign countries. But to this city is the loss most felt. Here was his lifework, and here in thousands of homes are those who will mourn his loss and cherish his memory. His name will long live as motive and inspiration to his professional brethren, here and elsewhere.

To this city he gave much, and it was appreciated; from it he received much, and it was appreciated, and for his life and work this community is the richer. His untimely end, the victim of a disease on which he had bestowed so much study, and from which he had rescued so many, seems the very irony of fate, and demonstrates the fact that the problems to which he had given so much thought and investigation have not yet reached their final analysis. An oft-repeated saying of Dr. Fowler that it was "as natural to die as to be born" has one more demonstration.

He was not so much of a scientist as to ignore the demands of the State upon him. He was never wanting in giving moral and substantial support to those measures and interests which fall to the common lot. His military services in the National Guard of this commonwealth, and for the United States in the Spanish war, are in keeping with such a spirit, to which he brought a zeal and efficiency of the highest order.

His spirit of self-sacrifice, his love of his profession and his unswerving loyalty to his friends, were dominant elements in his character.

The end came unexpectedly, with his mental and physical powers at their best. But his work is done, and well done, and the lessons of his life will be to those who remain a precious legacy and a potent inspiration.—*Brooklyn Standard Union.*

---

Splenic Leukemia.—H. J. Thompson reports the case of a teacher, thirty years old and single. Her past history did not show any severe illness. In December, 1901, she had "nervous spasms," the result of overwork, and shock, occasioned by the sudden death of her sister. In December of the same year she awakened one morning and found that she had lost the use of her limbs, and was in bed three weeks. Later on she regained the use of her limbs a little, but was very weak, with a pain in her left side, and soon after observed a growth on that side of the abdomen. In December, 1903, the skin was very dark in color over the abdomen; the menstruation had stopped several months before, and the kidneys were sluggish. The first Roentgen-ray treatment was given September 10, and continued once in five days until the end of the year. The menses reappeared immediately afterwards. All the treatments were given in a recumbent position, the patient being unable to sit up; a moderately hard tube was used, over the splenic region only, for fifteen minutes. As the treatments progressed, the symptoms abated, and soon after the patient returned home. In April she was able to walk without assistance, she gained flesh, and the menstrual periods were regular. From April until June she received three Roentgen-ray treatments a week. She returned home in June, lived out of doors all summer, and improved sufficiently to assist with the housework. Her weight increased to one hundred and thirty-seven pounds, and her waistline was reduced so that she could wear a blouse and belt, instead of a loose gown.—*Medical Record.*

